# Sociality deficits in serine racemase knockout mice

**DOI:** 10.1002/brb3.1383

**Published:** 2019-09-12

**Authors:** Tatyana M. Matveeva, Marc T. Pisansky, Amy Young, Robert F. Miller, Jonathan C. Gewirtz

**Affiliations:** ^1^ Department of Psychology University of Minnesota – Twin Cities Minneapolis Minnesota; ^2^ Department of Neuroscience University of Minnesota – Twin Cities Minneapolis Minnesota

**Keywords:** affect, asociality, glutamate, N‐methyl‐D‐aspartate, schizophrenia, sociability

## Abstract

**Background:**

Studies of schizophrenia have pointed to the role of glutamate in its pathophysiology. Mice lacking D‐serine show impairments in neurotransmission through NMDA receptors and display behaviors consistent with features of schizophrenia. Yet, socio‐communicative deficits, a characteristic of schizophrenia, have not been reported in serine racemase knockout mice.

**Methods:**

We use behavioral testing (the three‐chambered social approach task, the dyadic interaction task, and the novel object recognition task) to examine socio‐communicative behaviors in these mice.

**Results:**

Serine racemase mice show abnormal social investigation and approach behavior, and differ from wild‐type controls in the duration and number of vocalizations they emit in the presence of a conspecific. Serine racemase knockout mice were not impaired in a cognitive test (novel object recognition), although they displayed abnormal behavior in the acquisition phase of the task.

**Conclusions:**

Serine racemase knockout mice demonstrate abnormalities in socio‐communicative behaviors consistent with an impairment in sociality, a negative symptom of schizophrenia.

## Graphical abstract

Studies of schizophrenia have pointed to the role of glutamate in its pathophysiology. Mice lacking D‐serine show impairments in neurotransmission through NMDA receptors and display behaviors consistent with features of schizophrenia. Yet, socio‐communicative deficits, a characteristic of schizophrenia, have not been reported in serine racemase knockout mice. Our findings indicate that these mice show abnormal social investigation and approach behavior.

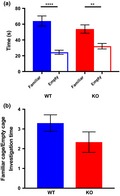

## 1 INTRODUCTION

Schizophrenia is a debilitating psychiatric disorder, marked by positive (e.g., hallucinations and delusions), negative (e.g., social withdrawal and deficits in motivation), and cognitive (e.g., deficits in working memory and verbal ability) symptoms. Characterizing changes in glutamatergic neurotransmission has been a major emphasis in efforts to elucidate this disease's underlying pathophysiology (Abi‐Saab, D'Souza, Moghaddam, & Krystal, 1998; Anand et al., 2000; Coyle, 2006; Gaisler‐Salomon, Diamant, Rubin, & Weiner, 2007; Lahti, Koffel, LaPorte, & Tamminga, 1995; Lahti, Weiler, Tamara Michaelidis, Parwani, & Tamminga, 2001; Tamminga, 1998; Zylberman, Javitt, & Zukin, 1995). Studies in humans and animals suggest a role for NMDA receptor hypofunction in the manifestation of endophenotypes related to schizophrenia's negative symptoms, including abnormalities in attention, affect, cognition, and social interaction (Blackman, Macdonald, & Chafee, 2013; Bubeníková‐Valesová, Horácek, Vrajová, & Höschl, 2008; Geyer, Krebs‐Thomson, Braff, & Swerdlow, 2001; Hardingham & Do, 2016; Linn, O'Keeffe, Lifshitz, Schroeder, & Javitt, 2007).

Depletion of D‐serine, an endogenous co‐agonist acting on the NMDAR's glycine site, has been proposed as an effective way to recapitulate NMDA receptor hypofunction in mice (Balu, Basu, Corradi, Cacace, & Coyle, 2012). The availability of D‐serine is dependent on the activity of the enzyme serine racemase. Serine racemase knockout mice (SRKOs) show a 90% reduction in cortical D‐serine resulting in substantially impaired NMDAR function (Basu et al., 2009). SRKOs also exhibit several cognitive and behavioral abnormalities consistent with schizophrenia (Balu et al., 2013). While evidence of the efficacy of glutamatergic agents for the treatment of negative symptoms remains mixed (Buchanan et al., 2017), some human studies report an amelioration of negative and positive and cognitive symptoms, in at‐risk populations after D‐serine treatment (Fujita, Ishima, & Hashimoto, 2016; Kantrowitz et al., 2010; Stip & Trudeau, 2005). Together, these findings suggest that low levels of D‐serine may contribute to schizophrenic symptomatology via a reduction in NMDA receptor function.

Asociality is a prominent negative symptom of schizophrenia, encompassing withdrawal from social interactions, avoidance of social environments, and absence or reduction of interest in forming relationships with others (Strauss & Cohen, 2017). Asociality thus has far‐reaching detrimental effects on daily functioning (Green, Hellemann, Horan, Lee, & Wynn, 2006). In contrast to the aforementioned findings, evidence for socio‐emotional abnormalities in these mice is lacking (DeVito et al., 2011). Here, we present evidence of aberrant social behavior in SRKO mice, including reduced social approach and social investigation, and changes in patterns of ultrasonic vocalizations. In contrast, cognitive function, measured using the novel object recognition task, was intact.

## 2 MATERIALS AND METHODS

### 2.1 Animals & genotyping

Mice were bred and maintained according to US National Institutes of Health guidelines for animal care and use and the Institutional Animal Care and Use Committee (IACUC) of the University of Minnesota—Twin Cities. Mice were provided food and water ad libitum except during experimental testing, and housing lights were maintained on a 12:12 hr light/dark cycle. All experimental mice were pair‐housed with same‐sex littermates. SR knockout mice were obtained via the Coyle Lab at Harvard University (Basu et al., 2009). Mice were genotyped using the following primers: 5′‐TGTGTGCTCAGTACTGCATCTCCT‐3′ (WT‐F); 5′‐TGGGCAACCTTATCCCTGATCCTT‐3′ (WT‐R), 5′‐GAATTTCCTCCTGTTAAGTGAATCTTCC‐3′ (KO‐F), 5′‐ACGTGGGAACCTGCTGGATTCT‐3′ (KO‐R). Different groups of mice were used as subjects in each behavioral paradigm.

### 2.2 Three‐chambered social approach

The three‐chambered social approach task has been used frequently to assess sociability and social approach in mice (Yang, Silverman, & Crawley, 2011). In this experiment, animals underwent three 10‐min phases within an arena (20 × 25 × 45 cm) partitioned into three chambers. In the first (habituation) phase, individual mice were allowed to explore the arena freely. In the second (sociability) phase, an age‐ and sex‐matched conspecific was enclosed under a small wire mesh cage (cup) within one chamber (hereafter referred to as the “mouse” chamber), while the other chamber contained an empty cup. The side of the apparatus containing the conspecific was randomized. In the third phase of the task, a novel conspecific was enclosed under a wire mesh cup on the opposite side of the chamber. For each phase, the times spent within each chamber and investigating each wire cage were scored by a genotype‐naïve (blind) experimenter. The ratio of time spent in the novel versus familiar chamber was calculated during the novelty phase.

### 2.3 Juvenile dyadic interaction

Postweaning (Postnatal Day 21) mice were handled for 3 consecutive days (5 min per day). Test mice (WT or KO) were paired with sibling heterozygous mice. On the test day, each mouse pair was placed into a 20 × 25 × 45 cm arena containing corncob bedding with a nontransparent partition for 5 min. Following removal of the partition, the mice were allowed to interact freely for 10 min. Oral–oral and oral–anogenital interactions were scored manually for each mouse by a genotype‐naïve experimenter using Button Box 5.0 (Behavioral Research Solutions, LLC). Ultrasonic vocalizations (USVs) were also recorded during interactions using high‐frequency microphones and Avisoft‐Recorder software (Avisoft Bioacoustics, Inc.).

### 2.4 Novel object recognition

Novel object recognition (NOR) test procedures were similar to those described previously by our laboratory (Pisansky et al., 2017) and others (Paban, Chambon, Jaffard, & Alescio‐Lautier, 2005). Testing comprised acquisition and test phases. Both were video‐recorded. Prior to each phase, animals were habituated to the test chamber for 1 min. Mice were then removed, objects were placed in the chamber, and the animals were returned to the chamber. During the acquisition phase, two identical objects were placed approximately 5 cm away from the opposite walls of the chamber and mice were allowed to explore freely for 10 min. At the end of the trial, mice were removed and then returned to the chamber for the test phase 1 hr later.

During the test phase, the familiar object from the acquisition phase was placed in one location within the chamber and a novel object was placed in the opposite location. Animals were placed in the test chamber containing both objects for 5 min. Upon completion of the test phase, mice were returned to their home cages. The test chamber was thoroughly wiped down with 70% alcohol, and objects were cleaned with diluted bleach between sessions.

The test phase made use of three different pairs of novel and familiar objects (for a total of six objects), which varied in color, material, texture, and shape. The location of the novel object in the test chamber, as well as the test order and the novel/familiar object pair, were counterbalanced across conditions. Object investigation during testing was scored by trained group‐naive raters using Button Box (Behavioral Research Solutions, LLC). During the acquisition phase of the task, time spent exploring either of the two identical objects placed in the testing apparatus was recorded. The total duration of investigation was calculated for each mouse (exploration score). During the test phase, a normalized novelty score was calculated for each animal by dividing the difference in investigation times for the novel and familiar objects by the time spent investigating the familiar object.

Preference for the novel object (and memory for the familiar object) was defined as time spent investigating the unfamiliar object compared to chance during the test phase.

### 2.5 Statistical analysis

Ultrasonic vocalizations were analyzed with custom‐built R script (a gift from Michael Saxe and Yan‐Ping Zhang Scharer, Roche). Generation and processing of spectrograms were conducted as described previously (Pisansky et al., 2017). USV frequency and duration were used to categorize each syllable into one of the following classes: NS, no frequency modulation (jumps), short duration (shorter than 25 msec); NL, no jumps, long duration (equal to or longer than 25 msec); JU, frequency jump upward (>7 kHz); MJ, multiple jumps. Statistical analyses of behavior in the NOR, dyadic interaction, and three‐chambered social approach tasks were conducted using R to examine group differences. *T* tests were used for analyses of behavioral data.

## 3 RESULTS

### 3.1 Three‐chamber test

**Figure 1:**
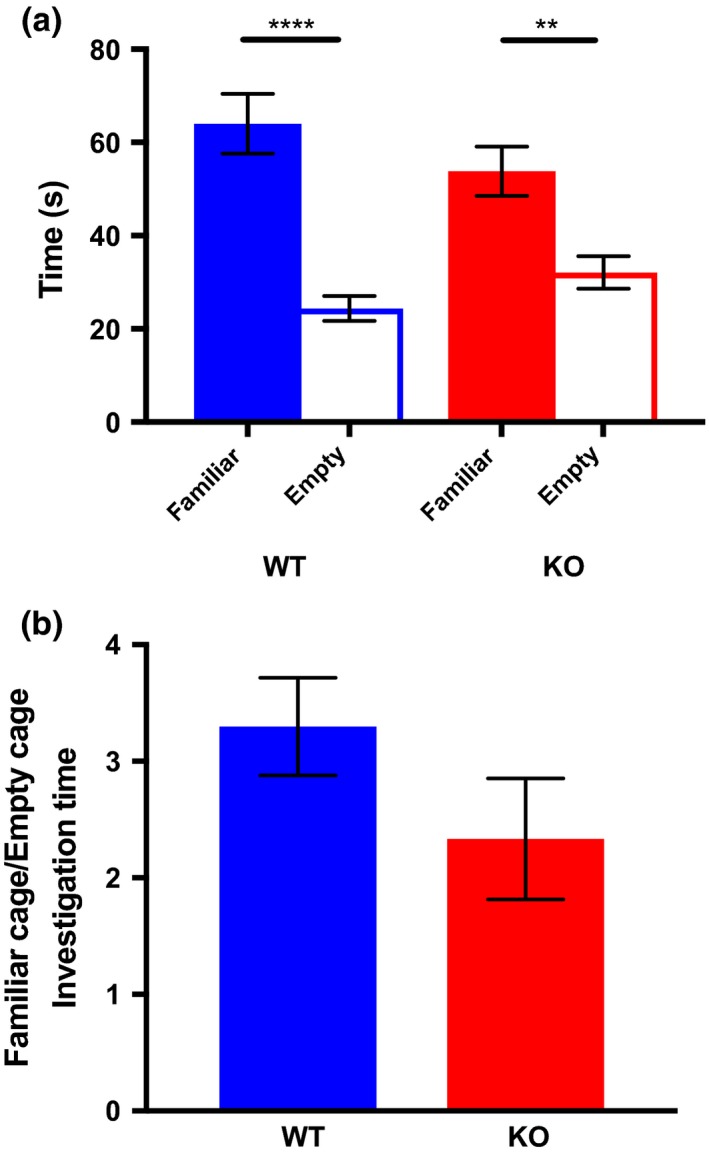
(a) Within‐group comparisons of time spent investigating the cage containing a familiar conspecific versus the empty cage during the sociability phase of the three‐chambered sociability task. Both WT (blue panels) and SRKO animals (red panels) spent more time investigating the familiar cage, though the preference was stronger in the WT group. Bars represent *SEM*. (b) Ratio of time spent investigating the familiar versus empty cage during the sociability phase of the three‐chambered social approach task. WT (blue panel) and SRKO (red panel) did not differ significantly in familiar to empty ratio. Bars represent *SEM*. ** denotes p<.01,*** denotes p<.001

In the initial phase of the task, both groups preferred the mouse chamber to the empty one (Figure 1a), although this preference was more pronounced in WT (*t* = 5.835, *df* = 25, *p* < .0001) than in KO mice (*t* = 3.578 *df* = 19, *p* < .01). There were no significant differences between the groups in the ratio of time spent investigating the mouse versus empty chamber (Figure 1b).

**Figure 2:**
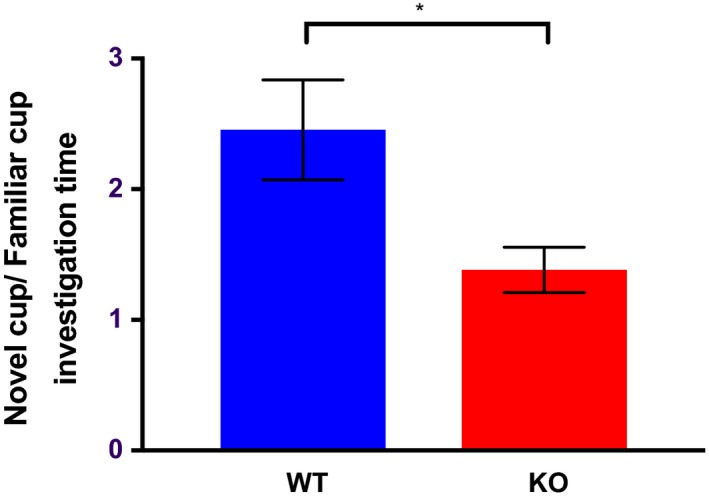
Between‐group comparisons of the ratio of time spent investigating the novel versus the familiar cup during the social novelty phase of the three‐chambered sociability task. KO, SR knockout mice; WT, wild‐type mice. Bars represent *SEM*. * denotes p<.05

During the social novelty phase, within‐group comparisons revealed a strong preference by WT mice for the chamber containing the familiar conspecific (*t* = 2.688, *df* = 25, *p* = .0126). The SRKO group did not show this preference (*t* = 0.1819, *df* = 20, *p* = .858). Further analysis indicated no between‐group differences in time spent exploring the novel chamber (*t* = 1.169, *df* = 45, *p* = .249), but a significant difference in the novel/familiar ratio (Figure 2), indicating that KO mice spent a greater fraction of time investigating the cup containing the familiar versus novel animal in comparison to controls (*t* = 2.361, *df* = 45, *p* = .022). Thus, SRKO mice demonstrated decreased novelty‐related exploration in social contexts.

### 3.2 Dyadic interaction

**Figure 3:**
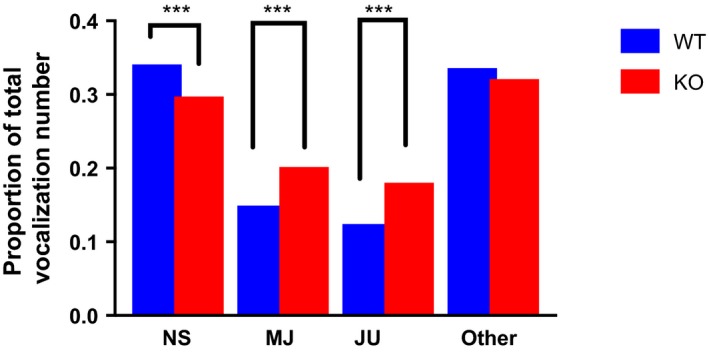
Between‐group comparisons for differences in the type of vocalization emissions measured as the proportion of total vocalization number. Blue bars = wild‐type controls, red bars = SR knockouts. NS = number of NS vocalizations/total number of vocalizations; MJ = number of MJ vocalizations/total number of vocalizations; JU = number of JU vocalizations/total number of vocalizations. *** denotes p<.001

Analyses revealed no differences in oral–oral and oral–anogenital investigation between WT and KO mice. However, a significant between‐group difference was revealed in the duration of vocalizations. Overall, SRKO animals showed longer total duration of vocalizations than WT mice (*t* = 2.0581, *df* = 53.599, *p* < .05). To further investigate whether there was a difference in vocalization type between the two groups, we derived normalized vocalization estimates for each syllable. For this analysis, we first calculated the frequency of occurrence of each syllable within each experimental group and divided this number by the total number of vocalizations within the group. The resulting measures were used in between‐group comparisons of vocalization types (Figure 3). In comparison to wild‐type controls, SRKO animals emitted a smaller proportion of NS (*χ* ^2^ = 26.253, *df* = 1, *p*‐value < .001) vocalizations, and a higher proportion of JU (*χ* ^2^ = 70.501, *df* = 1, *p*‐value < .001) and MJ vocalizations (*χ* ^2^ = 55.179, *df* = 1, *p*‐value < .001).

### 3.3 Novel object recognition

**Figure 4:**
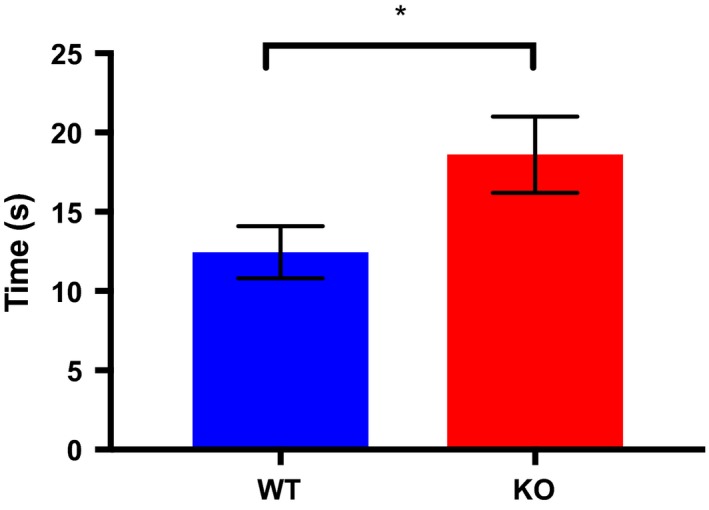
Exploration of the two identical objects during the acquisition phase of NOR for wild (WT), in blue, and SR knockout (KO), in red, animals. Bars represent *SEM*. * denotes p<.05

There were significant between‐group differences in exploration scores (Figure 4), with SRKO animals spending more time investigating the two identical objects during the acquisition phase than WT mice (*t* = 1.8435, *df* = 12.498, *p* = .04). There were no significant differences between the groups in the animals' relative exploration of the novel versus familiar object in the test phase (*t* = 0.61167, *df* = 10.901, *p* = .28).

## 4 DISCUSSION

Social deficits are a prominent negative symptom of schizophrenia and have a profound impact on quality of life. Given the putative relevance of NMDARs to the pathophysiology of the disorder, their contribution to aberrant social behavior has been studied extensively in rodent models utilizing pharmacological interventions and genetically modified animals (DeVito et al., 2011; Duncan et al., 2004; Mohn, Gainetdinov, Caron, & Koller, 1999; Panksepp et al., 2007). The present work investigated social interactions in mice when NMDA receptor transmission is disrupted indirectly and in a manner likely relevant to the etiology of schizophrenia (Zou et al., 2008), through deletion of the serine racemase gene. To accomplish this, we utilized two complementary behavioral paradigms: the three‐chambered social approach test and the dyadic interaction test. During the latter test, we also assessed USVs, a measure of socio‐emotional communication in rodents (Lahvis, Alleva, & Scattoni, 2011). Finally, we also conducted the novel object recognition task to test for deficits in cognitive function.

Serine racemase knockout animals displayed reduced motivation to seek interactions with conspecifics—a phenotype consistent with asociality. In the three‐chambered social approach test, SRKO mice failed to show a preference for the chamber containing the novel conspecific compared to the chamber containing the familiar animal and spent a larger fraction of total time in the latter chamber than did WT mice. This finding demonstrates reduced novelty‐related social exploration and is consistent with previous studies reporting reduced social interaction and increased social withdrawal in rodents following the administration of NMDA antagonists (Becker et al., 2003; Matsuoka et al., 2008; Panksepp et al., 2007; Uribe, Landaeta, Wix, & Eblen, 2013). Similar evidence for increased social withdrawal and decreased social exploratory behavior in response to NMDA blockade has also been observed in human subjects (Krystal et al., 1994; Lahti, Holcomb, Medoff, & Tamminga, 1995). Given the function of D‐serine as an NMDA receptor co‐agonist, our findings provide support for the contribution of reduced NMDAR activity in producing impoverished social behavior (Wilson & Koenig, 2014).

Interestingly, oral–oral and oral–anogenital interactions did not differ in WT and KO animals during the dyadic interaction task. However, in the same task, the SRKO mice emitted ultrasonic vocalizations that were fewer in number but of greater duration than those emitted by WT controls. The difference in vocalization duration was attributable to a greater proportion of frequency‐modulated vocalizations (JU and MJ subtypes) in the SRKO mice. Conversely, the same mice showed a lower proportion of short, nonmodulated vocalizations (NS). Although our understanding of the functional significance of specific patterns of USVs that are prototypical in the mouse is limited, it is interesting to note that variations in these patterns and their relative durations are related to sociality and subject to genetic influences (Lahvis et al., 2011). Furthermore, the relatively asocial B6 mouse strain shows longer USV durations than the BALB/c mouse during adolescence, similar to the pattern exhibited by the SRKO mice in the current study (Panksepp et al., 2007). While altered USV patterns have been found in mice harboring mutations of genes linked to autism spectrum disorders (Pisansky et al., 2017; Wöhr, 2014; Yang et al., 2015)., the current study is the first to report such an effect in a mutant mouse that recapitulates schizophrenia‐like behavior (Moy et al., 2007; Sungur, Stemmler, Wöhr, & Rust, 2018).

In the acquisition phase of the Novel Object Recognition (NOR) task, the SRKO mice exhibited greater exploration of the two identical objects. This may be due to an inability to properly form a stable representation in memory of the previously viewed object, or possibly indicative of increased perseverative behavior. In contrast, the SRKO mice did not show deficits in discriminating between the novel and familiar objects during the test phase of the task. This suggests a sparing of long‐term object recognition memory in these mice, consistent with previous findings (DeVito et al., 2011).

The fact that novel object preferences were intact in the SRKO mice is in itself an important positive control because it confirms that the observed abnormalities in their preferences for a novel conspecific were not due to a deficit in memory consolidation or to a more general aversion to novelty. Rather, the aberrant social behavior seen in both the social approach task and dyadic interaction suggests that SRKO animals manifest a pattern of abnormal social interaction akin to asociality—a prominent negative symptom of schizophrenia. This reinforces the view that depletion of D‐serine recapitulates a range of seemingly disparate schizophrenia‐like behavioral abnormalities in mice (Balu et al., 2013; Ma et al., 2013). Furthermore, our data are consistent with other findings linking NMDAR hypofunction to socially aberrant behavior in rodent models. For instance, NR1 hypomorphic mice display abnormalities in preference for social novelty, increased social avoidance, reduced social investigation, and suppression of sexual behavior, deficits that are attenuated by clozapine (Duncan et al., 2004; Mohn et al., 1999). Similarly, antagonism of NMDARs via administration of (+)‐MK‐801, ketamine, or memantine reduces social interaction in rats and diminishes social investigative behaviors in mice (Zou et al., 2008).

Thus, our findings lend support for the use of SRKO mice in studying mechanisms underlying impaired social functioning in schizophrenia and highlight the potential benefit of exploring the therapeutic utility of SR agonists in the treatment of this disorder's negative symptoms.

## CONFLICT OF INTEREST

None declared.

## Data Availability

The data that support the findings of this study are available from the corresponding author upon reasonable request.
